# Emergence of *bla*_*NDM–* 1_-Carrying *Aeromonas caviae* K433 Isolated From Patient With Community-Acquired Pneumonia

**DOI:** 10.3389/fmicb.2022.825389

**Published:** 2022-05-19

**Authors:** Xinhua Luo, Kai Mu, Yujie Zhao, Jin Zhang, Ying Qu, Dakang Hu, Yifan Jia, Piaopiao Dai, Jian Weng, Dongguo Wang, Lianhua Yu

**Affiliations:** ^1^Department of Clinical Laboratory Medicine, Taizhou Municipal Hospital Affiliated With Taizhou University, Taizhou, China; ^2^Beijing Institute of Radiation Medicine, Beijing, China; ^3^Beijing Key Laboratory of New Molecular Diagnosis Technologies for Infectious Diseases, Beijing, China; ^4^Department of Clinical Laboratory Medicine, Ningbo Medical Center Li Huili Hospital, Ningbo, China; ^5^Taizhou Center for Disease Control and Prevention, Taizhou, China; ^6^Department of Central Laboratory, Taizhou Municipal Hospital Affiliated With Taizhou University, Taizhou, China

**Keywords:** *Aeromonas* spp., IMEs, mobile genetic elements, *bla*_NDM_, multidrug resistance

## Abstract

To demonstrate the detailed genetic characteristics of a *bla*_NDM–1_-carrying multidrug-resistant *Aeromonas caviae* strain, the complete genome of the *A. caviae* strain K433 was sequenced by Illumina HiSeq and Oxford nanopore platforms, and mobile genetic elements associated with antibiotic resistance genes were analyzed by a series of bioinformatics methods. *A. caviae* K433 which was determined to produce class B carbapenemase, was resistant to most antibiotics tested except amikacin. The genome of K433 consisted of a chromosome cK433 (6,482-kb length) and two plasmids: pK433-qnrS (7.212-kb length) and pK433-NDM (200.855-kb length), the last being the first investigated *bla*_NDM_-carrying plasmid from *Aeromonas* spp. By comparison of the backbone and MDR regions from the plasmids studied, they involved a highly homologous sequence structure. This study provides in-depth genetic insights into the plasmids integrated with *bla*_NDM_-carrying genetic elements from *Aeromonas* spp.

## Introduction

*Aeromonas* spp. was first recognized as a human pathogen in 1954 when it was isolated from a blood sample (Parker and Shaw, 2011). In the following years, there were more confirmed cases of *Aeromonas* spp. causing human infections with varying degrees of severity, mainly, gastroenteritis (Parker and Shaw, 2011). *Aeromonas* spp. is ubiquitous in water, which can form biofilms, and then colonize the water system, drinking water may be a potential source of infection. *Aeromonas* spp. was mainly found in marine environments and freshwater (Figueira et al., 2011; Martino et al., 2014), and their spread is related to contact and ingestion of contaminated water or food.

In 2018, *Aeromonas* spp. was investigated in a wastewater treatment plant effluent in Tokyo, Japan, and two strains harboring the *bla*_KPC–2_ gene were detected (Sekizuka et al., 2019). Besides KPC, *Aeromonas caviae* producing VIM was reported in an Israeli hospital in 2014 (Adler et al., 2014), *Aeromonas hydrophila* carrying GES-24 carbapenemase was discovered in 2018 from a hospitalized patient in Okinawa, Japan (Uechi et al., 2018), and *A. caviae* from India was confirmed to carry OXA-181-carbapenemase (Anandan et al., 2017). *Aeromonas* spp. simultaneously harboring *bla*_CTX–M–15_, *bla*_SHV–12_, *bla*_PER–1_, and *bla*_FOX–2_, was isolated from Adriatic Sea of Croatia (Maravić et al., 2013). In the past 10 years, carbapenemase-producing bacteria have been isolated from non-human sources, including the aquatic environment. The carbapenemase-producing bacteria from the aquatic environment are particularly susceptible to human activities. Bidirectional movement of the carbapenemase-producing bacteria between the aquatic environment and humans has been occurring all the time (Hammer-Dedet et al., 2020). The fewest members of metallo-beta-lactamases B2 are composed of different species of *Aeromonas*, such as *A*. *hydrophila*, *Aeromonas Veronii*, and *Serratia Fonticola*, named CphA, ImiS, and SFH-I in the literature, respectively (Mojica et al., 2022). Just due to the pooling of carbapenemase-producing *Aeromonas* spp. from water, acquired resistance genes appear from time-to-time in the clinic, which deserves attention.

Although the aforementioned genes involving carbapenemase or other beta-lactamase were reported in *Aeromonas* spp., *bla*_NDM_ has not been reported in *A. caviae* to date. Since the *bla*_NDM_ gene was first discovered in India in 2009 (Yong et al., 2009), it has rapidly spread all over the world (Dortet et al., 2014). Although *bla*_NDM_ was originally determined in a *Klebsiella pneumoniae* plasmid (Yong et al., 2009), it has also been reported in the recent years that *bla*_NDM_ has been found in the chromosomes of *Enterobacteriaceae* (Girlich et al., 2015; Shen et al., 2017; Sakamoto et al., 2018; Reynolds et al., 2019; Kong et al., 2020). The strains carrying metallo-beta-lactamases are capable of hydrolyzing all beta-lactam antibiotics except aztreonam, which has raised great concerns worldwide.

In this work, we first discovered a multidrug-resistant *A. caviae* strain carrying *bla*_NDM–1_. The whole genome of the strain was sequenced and the mobile genetic elements of the strain containing drug-resistant genes were thoroughly and genetically studied.

## Materials and Methods

### Bacterial Strain and *16S rRNA* Gene

*A. caviae* strain K433 was isolated from a patient’s sputum in the Taizhou Municipal Hospital affiliated with the Taizhou University of China in 2018. EC600 (highly resistant to rifampicin) and *Escherichia coli* DH5α were used as hosts for conjugal and plasmid transfers, respectively. Strain K433 was initially identified by Vitek 2. Later, it was confirmed by PCR amplification and sequencing of *16S rRNA* with primers: Forward, 5′-AGAGTTTGATCATGGCTCAG-3′; Reverse: 5′-GGTTACCTTGTTACGACTT-3′ (Demarta et al., 1999). Moreover, bacterial species identification was also performed using genome sequence-based average nucleotide identity (ANI) analysis^1^ (Richter and Rosselló-Móra, 2009).

### Phenotypic Assays

#### Detection of Class a Serine Carbapenemase and Class B Metallo β-Lactamase

The activities of class A serine carbapenemase and class B metallo β-lactamase could be suppressed by 3-aminophenyl boronic acid (APB) and ethylenediamine tetra-acetic acid (EDTA) (Pournaras et al., 2013). We chose APB combined with EDTA to detect the carbapenemase of strain K433 according to the previous report (Tsakris et al., 2010).

The interpretation of the results was as follows: (1) if the diameter of the inhibition zone of the imipenem disc with APB solution differs from that of the single-imipenem disc by ≥5 mm, it could be judged that the tested strain produced class A carbapenemase; (2) if the diameter of the inhibition zone of the imipenem disc with EDTA solution differed from that of the single-imipenem disc by ≥5 mm, it might be that the tested strain produced class B carbapenemase; (3) If APB + EDTA were added concurrently, the diameters of the inhibition zone of the imipenem discs with APB + EDTA differed from that of the single-imipenem disc by ≥5 mm, it could be confirmed that the tested strain simultaneously produced class A carbapenemase + class B metallo β-lactamase; (4) if the difference between the inhibition zone diameter of the imipenem disc containing enzyme inhibitor and the single-imipenem disc was less than 5 mm, it could be determined that the bacteria did not produce class A carbapenemase or class B metallo β-lactamase.

#### Antibiotic Susceptibility Test

The method used for testing bacterial resistance was BioMérieux VITEK2, and the results were determined in accordance with the 2020 Clinical and Laboratory Standards Association (CLSI) guidelines (Clinical and Laboratory Standards Institute [CLSI], 2020).

12 antibiotics, namely, cefepime, aztreonam, imipenem, meropenem, amikacin, ciprofloxacin, levofloxacin, tigecycline, minocycline, tigecycline/clavulanic acid, and piperacillin/tazobactam, were tested. *E. coli* ATCC 25922 was used as the quality control strain.

### Conjugal Transfer and Plasmid Transfer

Bacterial plasmid DNA of strain K433 was extracted using a plasmid extraction kit (TaKaRa, Dalian, China) in accordance with the manufacturer’s instructions. The plasmid was transferred in an attempt from the *A. caviae* K433 isolate into EC600 and *E. coli* DH5a through conjugal transfer and electroporation, respectively. For the selection of transconjugants and/or transformants containing the *bla*_NDM_ marker, 2 μg/ml imipenem and 1,000 μg/ml rifampicin were used according to specific circumstances.

### Sequencing and Sequence Assembly

Genomic DNA was extracted from strain K433 using a Gentra Puregene Yeast/Bact. Kit (Qiagen, Valencia, CA, United States). Libraries were prepared separately using the TruePrepTM DNA Library Prep Kit V2 and the SQU-LSK109 Ligation Sequencing kit. After the preparation of the library was completed, it was separately sequenced on an Illumina HiSeq X Ten platform (Illumina Inc., San Diego, CA, United States) and GridION X5 platform (Oxford Nanopore, United Kingdom). To improve the reliability of data processing, raw data from the HiSeq X Ten platform and the GridION X5 platform were trimmed to obtain the high-quality clean reads (clean data) by Canu v1.8.^2^. The paired-end short Illumina reads and the long Nanopore reads were ‘‘*de novo*’’ assembled using Unicycler v0.4.5.^3^

### Sequence Annotation and Comparison

Open reading frames and pseudogenes were predicted using RAST2.0 (Brettin et al., 2015), BLASTP/BLASTN (Boratyn et al., 2013), UniProtKB/Swiss-Prot (Boutet et al., 2016), and RefSeq databases (O’Leary et al., 2016). Annotation of drug resistance genes, mobile genetic elements, and other features were performed using online databases, such as CARD (Liang et al., 2017), ResFinder (Zankari et al., 2012), ISfinder (Siguier et al., 2006), INTEGRALL (Moura et al., 2009), and the Tn Number Registry (Roberts et al., 2008). Multiple and pairwise sequence comparisons were performed using MUSCLE 3.8.31 (Edgar, 2004) and BLASTN. The genome map was drawn using Inkscape 0.48.1.^4^

### Nucleotide Sequence Accession Numbers

Nucleotide sequence accession numbers for chromosome K433 (ck433), plasmid K433-qnrS (pK433-qnrS), and plasmid K433-NDM (pK433-NDM) were CP084031, OK017455, and OK287926, respectively.

It was collected for comparative analysis between pK433-NDM and related plasmids, including p13ZX28-272, p13ZX28-TC-98, p13ZX36-200, pCP077202, pCP077203, and pCP077204, which nucleotide sequence accession numbers were MN101850, MN101852, MN101853, CP077202, CP077203, and CP077204, respectively.

## Results

### Antimicrobial Susceptibility Test, Enzymatic Properties, and Transferrable Features

Through the *16S rRNA* sequence and genome sequence-based ANI analysis, strain K433 was identified to be *A. caviae* eventually. The results of the antimicrobial susceptibility tests on strain K433 were shown in Table 1. Through detection of enzymatic properties, the strain K433 was confirmed to harbor only class B metallo β-lactamase. After bacterial conjugative transfer and electroporation assays, no transconjugant or transformant carrying pK433-NDM could be recovered despite repeated trials.

**TABLE 1:** Antimicrobial drug susceptibility profiles of *Aeromonas caviae* K433.

Antibiotics	MIC values (μg/mL)	Antimicrobial susceptibility
Ceftazidime	32	R
Cefepime	16	R
Aztreonam	16	R
imipenem	8	R
Meropenem	8	R
Amikacin	4	S
Ciprofloxacin	≥4	R
Levofloxacin	≥8	R
tigecycline	≥8	R
Minocycline	≥16	R
Ticarcilin/clavulanic acid	≥128	R
Piperacillin/tazobactam	≥128	R

### Overview of the Genome of K433

Strain K433 carried a 6,482-kb-long chromosome cK433, a 200.855-kb-long plasmid pK433-NDM, and a 7.212-kb-long plasmid pK433-qnrS (Supplementary Table 1). Plasmid pK433-NDM involved the region of *bla*_MOX–6_ gene, and a 42.3-kb-long MDR region where *bla*_NDM_ was inserted (Supplementary Figure 1). Plasmid pK433-qnrS only contained drug-resistance gene *qnrS2* (Supplementary Figure 2). All resistance genes were listed in Table 2.

**TABLE 2:** Resistance genes in the strain of K433.

Sequence	Resistance locus	Resistance phenotype	Nucleotide position	Region located
Chromosome K433	*aac(6’)-Ib-cr*	Fluoroquinolone and aminoglycoside resistance	1358857.1359456	IME1
	*arr3*	Rifampicin resistance	1359553.1360005	
	*bla*_TEM–1_	β-lactam resistance	1364218.1365078	
	*bla*_CTX–M–3_	β-lactam resistance	1365860.1366735	
	*sul1*	Sulfonamide resistance	1368946.1369785	
	*floR*	Phenicol resistance	1375626.1376840	
	*strA*	Aminoglycoside resistance	1384020.1384823	
	*strB*	Aminoglycoside resistance	1384823.1385659	
	*tetA(E)*	Tetracycline resistance	4241253.4242470	IME2
	*dfrA12*	Trimethoprim resistance	4646267.4646764	IME3
	*aadA2*	Aminoglycoside resistance	4647172.4647963	
	*qacED1*	Quaternary ammonium	4648127.4648474	
	*sul1*	Sulfonamide resistance	4648468.4649307	
	*chrA*	Chromate resistance	4649794.4650999	
	*mph(A)*	Macrolide resistance	4654618.4655523	
	*aphA-1*	Aminoglycoside resistance	4656500.4657315 4658360.4659175	
pK433-NDM	*bla*_MOX–6_	β-lactam resistance	57439.58587	*bla*_MOX–6_ region
	*mer locus*	Mercuric resistance	93469.97431	MDR region
	*mph(A)*	Macrolide resistance	100351.101256	
	*chrA*	Chromate resistance	104875.106080	
	*sul1*	Sulfonamide resistance	106567.107406 119294.120133	
	*bla*_OXA_	β-lactam resistance	107877.108671	
	*bla*_NDM–1_	β-lactam resistance	114445.115257	
	*ble*_MBL_	Bleomycin resistance	115261.115626	
	*qacED1*	Quaternary ammonium	120127.120474	
	*dfrA12*	Trimethoprim resistance	120981.121478	
	*aacC2*	Aminoglycoside resistance	123848.124708	
	*tmrB*	Tunicamycin resistance	124721.125263	
	*bla*_TEM–1_	β-lactam resistance	129680.130540	
pK433-qnrS	*qnrS2*	Quinolone resistance	2300.2956	–

### Characteristics of IMEs on Chromosome cK433

**FIGURE 1:**
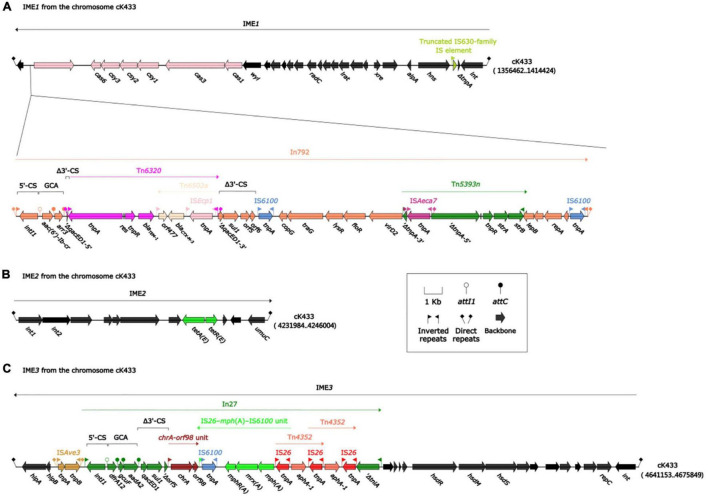
Mobile genetic elements associated with resistant genes on chromosome cK433. IMEs were abbreviation integrative and mobilizable elements. Three IMEs (IME*1*, IME*2*, and IME*3*) were to be discovered on chromosome cK433. **(A)** IME*1*, comprising the backbone region, *cas-csy* module and In792; **(B)** IME*2*, consisting of backbone region and *tetA-tetR* module; **(C)** IME*3*, involving the backbone region, IS*Ave3* and In27.

Integrative and mobilizable elements (IMEs) were extremely closely related to the acquisition or loss of bacterial resistance to antibiotics (Bellanger et al., 2014; Delavat et al., 2017). Three IMEs were found on cK433, including IME*1*, IME*2*, and IME*3* regions (Figure 1).

IME*1*, flanked by a pair of *attL/attR* (14 bp in length), had a backbone (containing *int*) with insertion of two accessory modules: 43.9-kb *strAB*–*bla*_CTX–M–3_ region and truncated IS*630*-family IS element. The 43.9-kb *strAB*–*bla*_CTX–M–3_ region, including In792 [gene cassette array (GCA): *aac(6’)-Ib-cr*–*arr3*], was inserted between the *orf339* and *wyl* gene at the left end of the backbone region, and truncated IS*630*-family IS element was inserted between the *hns* and *orf114* gene at the right end of the backbone region (Figure 1A). Meanwhile, the unit transposon Tn*6320* (carrying *bla*_TEM–1_ and *bla*_CTX–M–3_) was inserted into the *qacED1* gene of In792. Tn*5393n* was inserted between the *virD2* and *lepB* gene at the right end of In792, following, IS*Aeca7* was inserted into Δ*tnpA* gene, which was divided into two parts on the left end of Tn*5393n*, then, two identical IS*6100*s were inserted between the 3′-CS and the right end of In792, forming the current complex IME*1* structure just like “Russian nesting dolls.”

IME*2* consisted of the backbone region and *tetA-tetR* module which was related to tetracycline drug resistance (Figure 1B). IME*3* contained the backbone region, IS*Ave3* and In27 [GCA: *dfrA12*–*gcuF*–*aadA2*] which was truncated by *chrA-orf98* unit, IS*26-mph(A)*-IS*6100* unit, and two intersecting Tn*4352* (Figure 1C).

### Comparison of Plasmids pK433-NDM, pCP077202, pCP077203, and pCP077204

**FIGURE 2:**
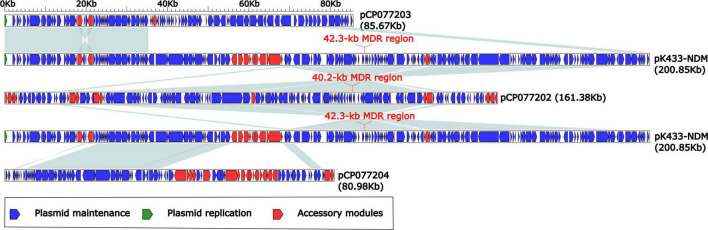
Comparison of plasmids pK433-NDM, pCP077202, pCP077203, and pCP077204. Plasmids pCP077202, pCP077203, and pCP077204 were obtained from GenBank, which came from *Aeromonas* spp. in the United States. Plasmids pCP077202, pCP077203, and pCP077204 had 161.381, 85.67, and 80.98 kb lengths, respectively. The shadow of light blue represented >95% identity.

**FIGURE 3:**
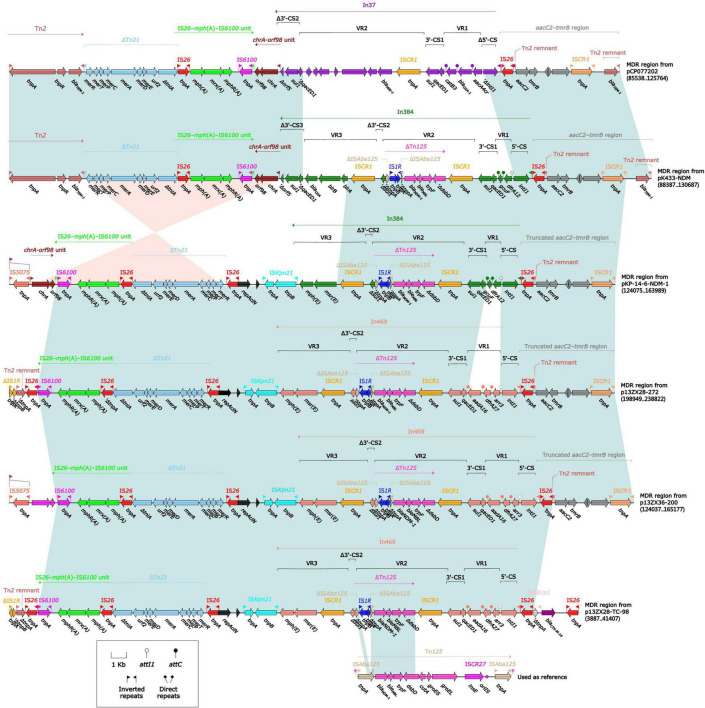
Comparison of MDR regions from plasmids pK433-NDM, pCO077202, pKP-14-6-NDM-1, p13ZX28-272, p13ZX36-200, and p13ZX28-TC-98. pK433-NDM and pCO077202 came from *Aeromonas* spp. pKP-14-6-NDM-1 was isolated from *Klebsiella pneumoniae*, and p13ZX28-272, p13ZX36-200, and p13ZX28-TC-98 were all achieved from *Escherichia coli*.

According to the BLASTN alignments of the complete sequence of plasmid pK433-NDM in the NCBI GenBank database, we found that the top three plasmids ranked by coverage value were pCP077202 (59%), pCP077203 (26%), and pCP077204 (24%), and their identities were both 100%. These three plasmids (pCP077202, pCP077203, and pCP077204) collected from GenBank all belonged to *Aeromonas* spp. in the United States and had a close correlation with plasmid pK433-NDM. Plasmids pCP077202, pCP077203, and pCP077204 were from the same strain with 161.381-, 85.67-, and 80.98-kb length, respectively. The sequence composition and structure of pK433-NDM and pCP077203 were highly similar (>95% identity) around the first 35 kb length in the plasmid maintenance region (Figure 2). Both plasmids pK433-NDM and pCP077202 contained the MDR region, in which there was also a high similarity with the composition and structure located on the MDR region upstream and downstream of the plasmid maintenance regions (>95% identity) (Figure 2). The comparison of MDR regions for 42.3 kb long pK433-NDM and 40.2 kb long pCP077202 is illustrated in Figure 3. The composition and structure of the sequence approximate 32 kb long on the left end of plasmid maintenance regions of pK433-NDM (23111.55424) and pCP077204 (5096.36653) were also highly similar (>95% identity). However, no plasmid replication gene was found in plasmid pCP077204 and pCP077202.

### Comparison of MDR Regions From Plasmids pK433-NDM, pCP077202, pKP-14-6-NDM-1, p13ZX28-272, p13ZX36-200, and p13ZX28-TC-98

All the aforementioned plasmids except pK433-NDM were obtained from GenBank. pKP-14-6-NDM-1 was isolated from *K. pneumoniae* and p13ZX28-272, p13ZX36-200, and p13ZX28-TC-98 were all achieved from *E. coli*. The coverage and identity of the MDR region from aforementioned plasmids were listed in Supplementary Table 2. Compared with MDR regions from plasmids pK433-NDM and pCP077202, it seemed that In37 [Variable region 1 (VR1) containing *aacA4cr*, *bla*_OXA–1_, and *catB1* and VR2 containing *bla*_PER–1_] of MDR region from pCP077202 was replaced by In384 [VR1 containing *dfrA12*, VR2 containing *bla*_NDM–1_ and *ble*_MBL_, and VR3 containing *bla*_OXA_] of MDR region from pK433-NDM, and the remaining regions had a high degree of identity (>95%) with MDR region from pK433-NDM. (Figure 3). However, the MDR region from pK433-NDM carried the *bla*_NDM–1_ gene located in the truncated composite transposon Tn*125*, while the MDR region from pCP077202 did not, which was the significant difference between them. Compared with MDR regions from pK433-NDM and pK-14-6-NDM-1, both involved *bla*_NDM_ gene and were highly consistent with *aaC2-tmrB* region and In384 (>95% identity), and also ΔTn*21*, *chrA-orf98* unit and IS*26-mph(A)*-IS*6100* unit (>95% identity). Compared with MDR regions from pK-14-6-NDM-1 and p13ZX28-272, the regions containing *bla*_NDM_ gene showed high consistency except for the insertion type of integron (In469 from p13ZX28-272 replaced by In384 from pK-14-6-NDM-1). Compared with p13ZX28-272, p13ZX36-200, and p13ZX28-TC-98, MDR regions of pK-14-6-NDM-1 and p13ZX28-272 showed the highest identity (>95%) but revealed different coverage, which was listed in Supplementary Table 2. Interestingly, In469 and In384 in pK433-NDM, pK-14-6-NDM-1, p13ZX28-272, p13ZX36-200, and p13ZX28-TC-98 all contained the identical IS*CR1* and ΔTn*125* structure, which suggested IS*CR1* prompted the accumulation of ΔTn*125* between these plasmids.

## Discussion

Various types of antibiotic resistance genes have been discovered over and over again in *Aeromonas* spp. from nature, which is commonly resistant to quinolone and β-lactam drugs (Piotrowska et al., 2017). It is very likely that *Aeromonas* spp. is naturally an important repository of acquired β-lactamase genes from wastewater or sludge, which was to be found in plentiful genes harboring classes A, B, C, and D β-lactamase (Piotrowska et al., 2017). However, only a tiny amount of class B carbapenemases were found, such as AsbM1, IMP-19, VIM, ImiS, ImiH, and CphA (Janda and Abbott, 2010; Piotrowska et al., 2017). So far, NDM had never been reported. To our knowledge, this is the first study involving NDM carbapenemase from an *A. caviae* strain (K433), which was isolated from inpatient’s source with multidrug-resistance in our hospital. This study not only provided the first evidence of nosocomial infection and colonization of an NDM-producing *A. caviae*, but also revealed the strong transmission ability of NDM.

Reported firstly in 2009, NDM-1 has caused a major public health problem because of its high resistance profile to carbapenems and its global prevalence (Yong et al., 2009). To date, 40 variants of NDM carbapenemases have been reported.^5^ The bacterial strains harboring *bla*_NDM_ exhibited significantly increased MICs for carbapenems, cephalosporins, penicillins, ticarcilin/clavulanic acid, and piperacillin/tazobactam except for aztreonam, just as shown in the susceptibility test of *A. caviae* K433 (Table 1). Strain K433 was resistant to almost all the antibiotics (including imipenem, meropenem, and tigecycline) except amikacin. Among the reported mechanisms of tigecycline resistance, the bacterial efflux pump system plays a major role. The overexpression of characteristic efflux pumps AdeABC, AdeFGH, and AdeIJK, together with the deletion and mutation of the two-component regulatory systems adeR and adeS, can lead to tigecycline resistance (Nguyen et al., 2014). In addition, the reasons for the decreased sensitivity to tigecycline include the inactivation of tigecycline by the modification enzyme Tet(X), the alteration of the cell membrane permeability because of the mutation of the *plsC* gene, and the decreased affinity between tigecycline and the ribosome due to the mutation of the *rpsJ* gene, etc. (Beabout et al., 2015). Recently, studies have reported that tigecycline resistance can be transmitted in bacteria by conjugation of plasmids carrying resistance genes (Partridge et al., 2018). In this study, we have not detected the *tet(X)* gene or other tigecycline resistance genes in strain K433. The possible mechanisms of tigecycline resistance in K433 were the overexpression of bacterial efflux pump system or/and the altering of cell membrane permeability, etc.

After high-throughput sequencing, it was determined that *A. caviae* K433 carried two plasmids: (pK433-NDM and pK433-qnrS), and one 6,482-kb-long chromosome cK433, carrying three IMEs: (IME*1*, IME*2*, and IME*3*) (Figure 1). IMEs and ICEs (integrative and conjugative elements) (Botelho and Schulenburg, 2021) are two different types of mobile genetic elements. They are often integrated into bacterial chromosomes to prompt the spread of resistance genes. IMEs cannot be self-transmitted, and they move between cells with the help of other conjugative elements that encode proteins involving in the complete conjugation function. IMEs usually have *attL*, *int*, *rlx*, *oriT*, and *attR*, but do not contain conjugative transfer genes (Luo et al., 2021). As for other properties of chromosome cK433, further study is needed.

It was utterly different between pK433-NDM and pK433-qnrS. Plasmid pK433-qnrS (7212 kb in length) had only *qnrS2-repC-repA-mob* gene cassettes (Supplementary Figure 2), while plasmid pK433-NDM (200.855 kb in length) possessed the backbone, including plasmid maintenance and replication regions, and variable regions: 42.3-kb MDR region, *bla*_MOX–6_ region, IS*Aeme19* and IS*AS17* (Supplementary Figure 1). We speculated that such a length of plasmid and the complex structure of the MDR region may result in the failures of plasmid conjugative transfer and electroporation experiment for pK433-NDM. There were six units or modules in the MDR region from pK433-NDM, revealing Tn*2*, ΔTn*21*, IS*26-mph(A)*-IS*6100* unit, *chA-orf98* unit, In384, and *aaC2-tmrB* region (Figure 3). The biggest differences between the MDR regions from pCP077202 and pK433-NDM were that In384 from pK433-NDM replaced the position of In37 from pCP077202, and, In37 involved 2 variable regions (VR), In384 contained 3 variable regions, then, VR2 carried ΔTn*125* with *bla*_NDM_ (Figure 3). Such a complex plasmid structure would greatly enhance the resistance to the drugs, such as carbapenems, cephalosporins, and penicillins (Table 1). Except that the MDR region was somewhat comparable, the backbone regions of plasmids: pCP077202, pCP077203, and pCP077204 which came from the same strain were more or less identified with the pK433-NDM, but there were some repeat backbone regions between the pCP077202, pCP077203, and pCP077204 (Figure 2). In general, these plasmids from different *Aeromonas* spp. had more similar structures and compositions despite of coming from different countries, different times, and even different races (Figure 2). As for the comparative analysis of the MDR regions from the pK433-NDM, p13ZX28-272, p13ZX28TC-98, pKP14-6-NDM-1, and p13ZX36-200, we found that the MDR regions from different plasmids almost had the identical structure, harboring ΔTn*125* with *bla*_NDM_ gene. It suggested that after the In384 carrying ΔTn*125* with *bla*_NDM_ gene was replaced by the In469 which also carried the ΔTn*125* with *bla*_NDM_ gene, it might be evolved even more epidemic; simultaneously, we also speculated that part of the plasmid structure and composition of *A. caviae* cK433 might come from other popular plasmids, and there was a potential risk of transmission, which must be actively prevented.

## Conclusion

This study characterized the genome structure and constitution of the *bla*_NDM_-carrying multidrug-resistant *A. caviae* strain K433. Plasmids pK433-NDM and pK433-qnrS and chromosome cK433 were discovered. In total, three drug-resistant-gene-associated IMEs (IME*1*, IME*2*, and IME*3*) were inserted into complex gene structures, including integrons, transposons, and other mobile genetic modules or units, and studied in cK433. Four plasmids: pK433-NDM, pCP077202, pCP077203, and pCP077204 were compared with the backbone and MDR regions. It showed a highly homologous sequence structure between pK433-NDM and plasmids from the same strain: pCP077202, pCP077203, and pCP077204, in the backbone regions. It also indicated a highly homologous sequence structure between the MDR regions of pK433-NDM, pCP077202, pKP-14-6-NDM-1, p13ZX28-272, p13ZX36-200, and p13ZX28-TC-98. This study would provide a further theoretical basis for genetic evolution for plasmids involving *bla*_NDM_-carrying genetic elements from *Aeromonas* spp.

## Data Availability Statement

The original contributions presented in the study are publicly available. This data can be found here: PRJNA765169, OK287926, and OK017455.

## Author Contributions

LY and DW conceptualized and designed the study. All authors participated and acquired the data. XL, KM, LY, and DW analyzed and interpreted the data. XL and KM drafted the manuscript. LY and DW critically revised the manuscript. All authors read and approved the final manuscript.

## Conflict of Interest

The authors declare that the research was conducted in the absence of any commercial or financial relationships that could be construed as a potential conflict of interest.

## Publisher’s Note

All claims expressed in this article are solely those of the authors and do not necessarily represent those of their affiliated organizations, or those of the publisher, the editors and the reviewers. Any product that may be evaluated in this article, or claim that may be made by its manufacturer, is not guaranteed or endorsed by the publisher.

## Footnotes

1
http://www.ezbiocloud.net/tools/ani

2
https://canu.readthedocs.io/en/latest/index.html

3
https://github.com/rrwick/Unicycler

4
https://inkscape.org/en

5
http://www.bldb.eu/Enzymes.php
