# Supplementary material for: Emergence of blaNDM– 1-Carrying Aeromonas caviae K433 Isolated From Patient With Community-Acquired Pneumonia
Source: Front Microbiol. 2022 May 19;13:825389. doi: 10.3389/fmicb.2022.825389 (PMC9161076; doi:10.3389/fmicb.2022.825389)
Supplement: Supplementary file 1 [file Data_Sheet_1.zip › Tables S1 and S2.docx]

**TABLE S1** Whole genome information of *Aeromonas caviae* K433

| **Sequence** | **Mean G+C content, %** | **Length (bp)** | **Total number of ORFs** | **MLST** | **Inc type** | **Accession**  **number** |
| --- | --- | --- | --- | --- | --- | --- |
| cK433 | 61.4% | 4,682,417 | 4,265 | ST141 | - | CP084031 |
| pK433-NDM | 55.0% | 200,855 | 201 | - | Unknown | OK287926 |
| pK433-qnrS | 59.9% | 7,212 | 6 | - | IncQ | OK017455 |

-, not available

| **TABLE S2 Comparison of coverage and identity in MDR regions from plasmids** | | | | | | |
| --- | --- | --- | --- | --- | --- | --- |
| MDR region | Coverage/Identity | | | | | |
|  | pK433-NDM | pCP077202 | pKP-14-6-NDM-1 | p13ZX28-272 | p13ZX36-200 | p13ZX28-TC-98 |
| MDR region from pK433-NDM | 100%/100% | 81%/99.98% | 77%/99.99% | 72%/99.72% | 75%/100% | 59%/100% |
| MDR region from pCP077202 | 81%/99.98% | 100%/100% | 62%/99.99% | 59%/99.71% | 62%/99.99% | 46%/99.99% |
| MDR region from pKP-14-6-NDM-1 | 77%/99.99% | 62%/99.99% | 100%/100% | 90%/99.84% | 97%/99.96% | 77%/99.95% |
| MDR region from p13ZX28-272 | 72%/99.72% | 59%/99.71% | 90%/99.84% | 100%/100% | 98%/99.91% | 86%/99.90% |
| MDR region from p13ZX36-200 | 75%/100% | 62%/99.99% | 97%/99.96% | 98%/99.91% | 100%/100% | 80%/99.99% |
| MDR region from p13ZX28-TC-98 | 59%/100% | 46%/99.99% | 77%/99.95% | 86%/99.90% | 80%/99.99% | 100%/100% |
