# Supplementary figures and images for: Emergence of blaNDM– 1-Carrying Aeromonas caviae K433 Isolated From Patient With Community-Acquired Pneumonia
Source: Front Microbiol. 2022 May 19;13:825389. doi: 10.3389/fmicb.2022.825389 (PMC9161076; doi:10.3389/fmicb.2022.825389)

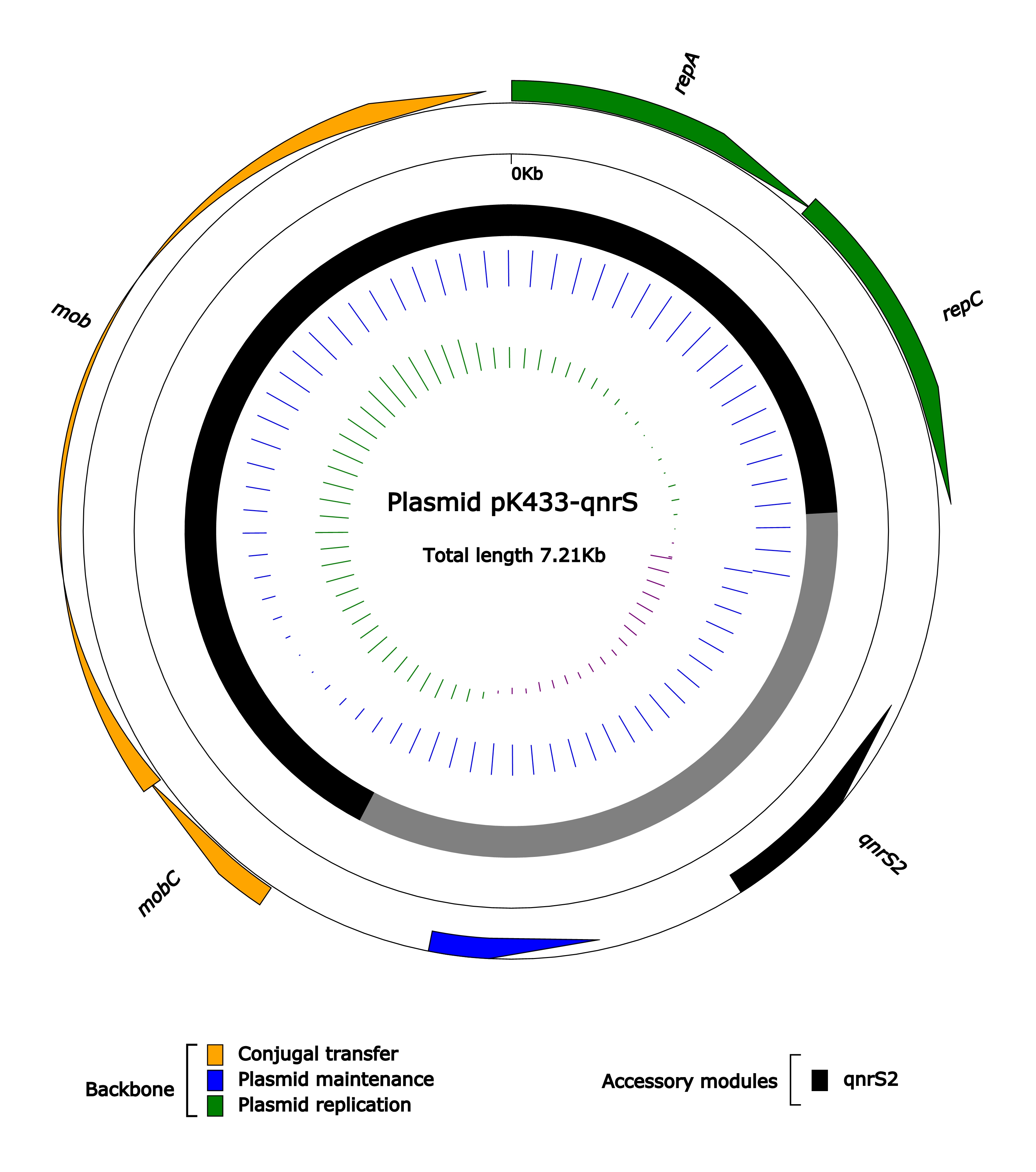

Supplement: Supplementary file 1 [file Data_Sheet_1.zip › FIGURE S2.tif]

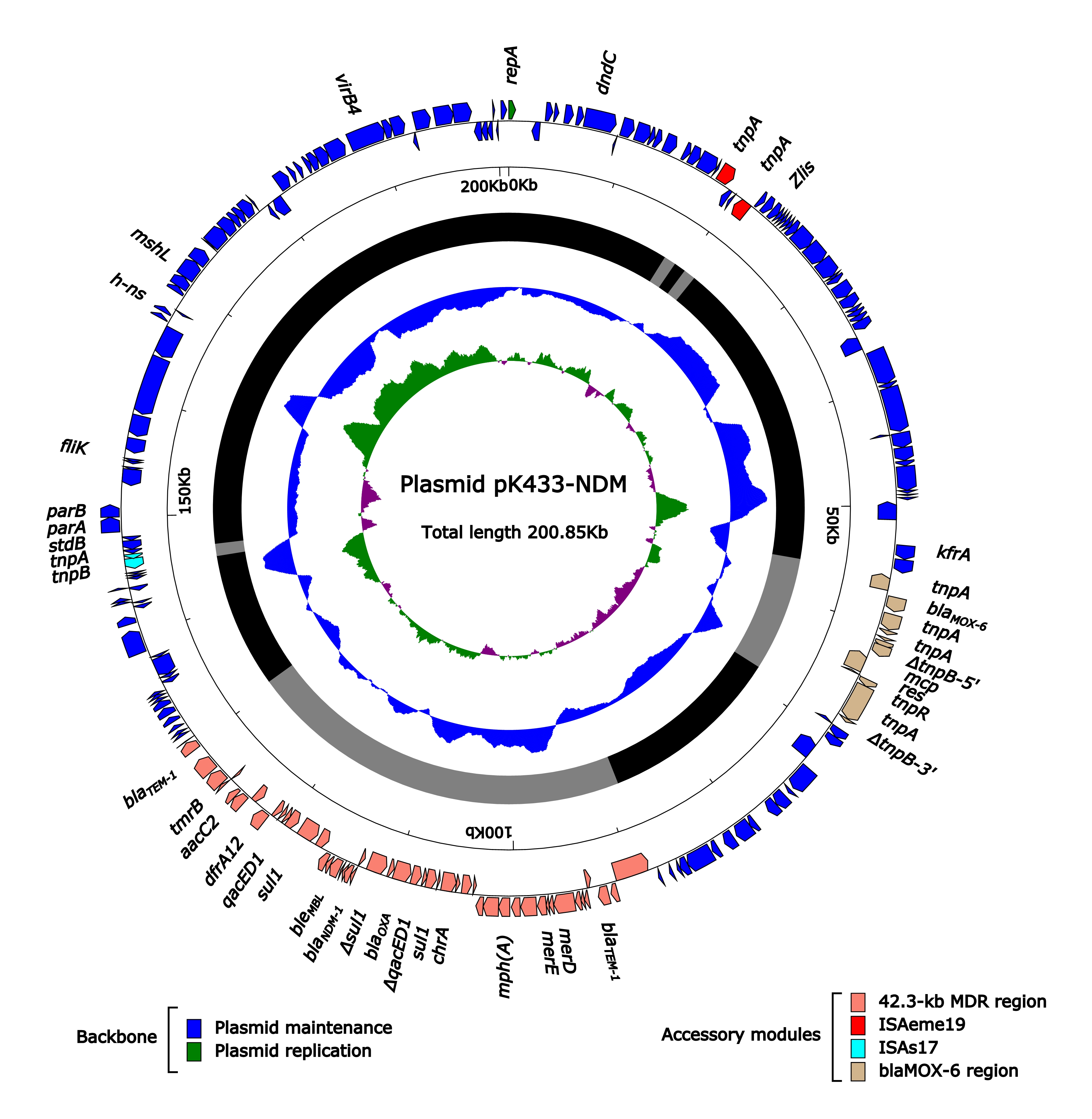

Supplement: Supplementary file 1 [file Data_Sheet_1.zip › FIGURE S1.tif]
